# Paired Helical Filament-Forming Region of Tau (297–391) Influences Endogenous Tau Protein and Accumulates in Acidic Compartments in Human Neuronal Cells

**DOI:** 10.1016/j.jmb.2020.05.027

**Published:** 2020-08-07

**Authors:** Saskia J. Pollack, Jasmine Trigg, Tahmida Khanom, Luca Biasetti, Karen E. Marshall, Youssra K. Al-Hilaly, Janet E. Rickard, Charles R. Harrington, Claude M. Wischik, Louise C. Serpell

**Affiliations:** 1 Sussex Neuroscience, School of Life Sciences, University of Sussex, Falmer, E. Sussex, BN1 9QG, UK; 2 Chemistry Department, College of Science, Mustansiriyah University, Baghdad, Iraq; 3 Institute of Medical Sciences, University of Aberdeen, Aberdeen, AB25 2ZP, UK; 4 TauRx Therapeutics Ltd., Aberdeen, AB24 5RP, UK

**Keywords:** tau, Alzheimer's disease, propagation, aggregation, AD, Alzheimer's disease, PHF, paired helical filament, TEM, transmission electron microscopy, CD, circular dichroism, DMEM/F-12, Dulbecco's modified Eagle medium/Nutrient Mixture F-12, P/S, penicillin/streptomycin, BSA, bovine serum albumin, PFA, paraformaldehyde

## Abstract

Assembly of tau protein into paired helical filaments and straight filaments is a key feature of Alzheimer's disease. Aggregation of tau has been implicated in neurodegeneration, cellular toxicity and the propagation, which accompanies disease progression. We have reported previously that a region of tau (297–391), referred to as dGAE, assembles spontaneously in physiological conditions to form paired helical filament-like fibres *in vitro* in the absence of additives such as heparin. This provides a valuable tool with which to explore the effects of tau in cell culture. Here we have studied the cellular uptake of soluble oligomeric and fibrillar forms of dGAE and examined the downstream consequences of tau internalisation into differentiated SH-SY5Y neuroblastoma cells using fluorescence and electron microscopy alongside structural and biochemical analyses. The assembled dGAE shows more acute cytotoxicity than the soluble, non-aggregated form. Conversely, the soluble form is much more readily internalised and, once within the cell, is able to associate with endogenous tau resulting in increased phosphorylation and aggregation of endogenous tau, which accumulates in lysosomal/endosomal compartments. It appears that soluble oligomeric forms are able to propagate tau pathology without being acutely toxic. The model system we have developed now permits the molecular mechanisms of propagation of tau pathology to be studied *in vitro* in a more physiological manner with a view to development of novel therapeutic approaches.

## Highlights

• Tau297–391 aggregates to form filaments toxic to cells in the absence of additives.
• Internalisation of soluble tau297–391 into cells leads to increased insoluble Ptau.
• Soluble tau297–391 accumulates with endogenous tau in endo-lysosomal compartments.

## Graphical abstract

**Figure:**
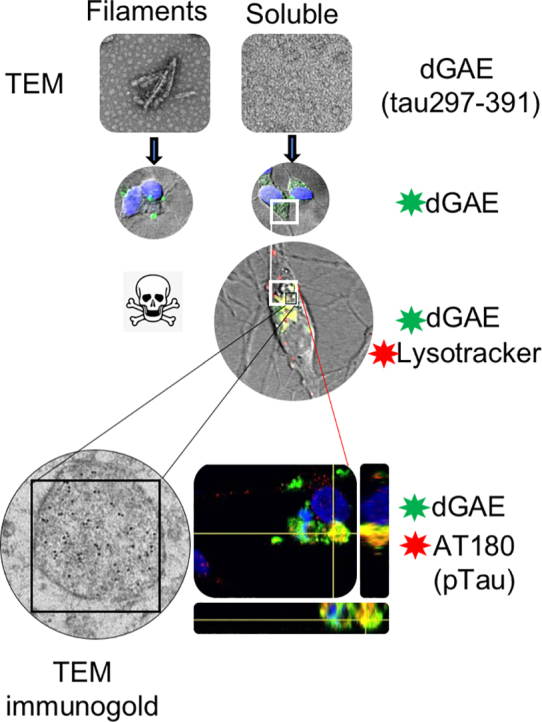

## Introduction

A common pathological process amongst neurodegenerative diseases is the accumulation of amyloid aggregates formed by disease-specific proteins in the cytoplasm, nucleus or extracellular space [1]. The misfolding, self-assembly and accumulation of tau protein in neurofibrillary tangles is a major pathological feature shared by tauopathies, the most common of which is Alzheimer's disease (AD). Under pathological conditions, the abnormal aggregation of tau protein into paired helical filaments (PHFs) that constitute the neurofibrillary tangles is accompanied by the loss of function of tau protein and neuronal dysfunction.

The identity of the species of tau that is most toxic has been debated [[2], [3], [4], [5]]. It was thought that neurofibrillary tangles are an integral feature of tau toxicity, since their number and distribution in the brain correlate with cognitive decline in AD [[6], [7], [8]]. However, as with other amyloidogenic proteins implicated in neurodegeneration, it has been proposed that tau oligomers are the toxic species responsible for the correlation between lesions and neurodegeneration [[9], [10], [11], [12], [13]]. Hypotheses regarding oligomeric tau toxicity have been suggested based on common mechanisms of toxicity shared amongst amyloid proteins, including mitochondrial dysfunction, increase in ion permeability of membranes, increased intracellular calcium, leakage of cellular contents, and dysfunction of the autophagy-lysosomal machinery [14,15].

Tau has been shown to exhibit prion-like propagation, including cellular uptake and templated seeding [[16], [17], [18], [19], [20], [21], [22]]. *In vitro* studies have shown that extracellular tau aggregates can be internalised by neurons to induce self-assembly of intracellular endogenous tau, which can then be released and transferred to neighbouring or synaptically connected neurons [[23], [24], [25], [26]]. In cultured cell lines, internalised aggregates of recombinant tau induced using heparin or AD brain-derived tau aggregates can be observed in endosomal compartments and are capable of recruiting endogenous and aggregation-prone tau to aggregate [16,19,23,[25], [26], [27], [28]]. A consistent finding in these studies is the colocalisation of tau with endosomes and lysosomes [19,23,24,26].

Whilst there is strong support for transmission of tau aggregates between neurons and/or glial cells, the species responsible for this has been debated. Studies have suggested that the seeding competence of tau is dependent on the size and conformation of the tau aggregate and that tau oligomers act as the key species for inducing propagation [13] rather than monomers or longer fibrils purified from rTg4510 mice [26]. It was reported in one study that large tau aggregates (> 10 mers) are the seed-competent species in P301S tau transgenic mice [29], whereas in another study, tau trimers were found to be the minimal unit necessary for conformational template seeding and intracellular tau aggregation in human tau-expressing HEK-293 cells [30].

Many studies have used animal or cell models in which human and/or mutant tau has been overexpressed. *In vitro* studies have utilised full-length or truncated tau proteins that require heparin-induced fibrillisation. Both approaches aim to overcome the low aggregation propensity and the lack of cytotoxicity of full-length tau [31]. However, there are increasing doubts as to the physiological relevance of heparin-induced tau filaments as they do not reproduce the key self-assembly or structural features of AD filaments [[32], [33], [34]]. The truncated repeat-domain fragment of tau spanning residues 297–391 (referred to as dGAE (see [[33], [34], [35], [36], [37]] for nomenclature)), which was first identified biochemically in proteolytically stable PHF core preparations from AD brain tissues [[35], [36], [37]], includes the core sequence (306–378) characterised by cryo-electron microscopy as forming C-shaped subunits assembled to form a combined cross-β/β-helix structure [38]. It is not understood what initiates the process of tau aggregation in AD, but truncated dGAE serves for template-directed aggregation of tau in cell-free and cellular models [22,39] and in transgenic mice [40]. In previous work, we have reported that under certain conditions, dGAE readily assembles into amyloid-like fibrils that are morphologically similar to native PHFs *in vitro* without the need for exogenous seeding factors [41,42]. This provides a useful model system with which to explore cellular effects of this core tau region in the form of soluble or fibrillar aggregates. Here we have developed the use of a fluorescently labelled version of dGAE to study its internalisation and downstream toxic effects in differentiated human neuroblastoma cells (dSH-SY5Y) expressing endogenous, full-length human tau at normal levels.

We report here that aggregated dGAE is cytotoxic whilst the soluble, non-fibrillar dGAE is not acutely toxic and is taken up by neuron-like cells. Internalisation results in production of insoluble tau species, abnormal phosphorylation and truncation of endogenous full-length tau. Immunofluorescence and immunogold transmission electron microscopy (TEM) of treated cells reveals that dGAE accumulates within the endosomal/lysosomal compartments.

## Materials and Methods

### Preparation of recombinant dGAE

Purified recombinant truncated tau (dGAE, corresponding to amino acid residues 297–391 using numbering from 2N4R tau) was used throughout the study. Recombinant tau protein 297–391 was purified as previously described [41]. Following purification, dGAE exists in a predominantly random coil conformation and consists mainly of soluble monomer and dimer as previously characterised and described [41].

### Alexa Fluor® 488 labelling of tau protein

To generate fluorescently tagged tau protein (dGAE-488), dGAE was covalently labelled with Alexa Fluor 488® (Life Technologies) by mixing 200 μl protein (425.2 μM) with 10 μl 113 nM Alexa Fluor® TFP ester and 20 μl 1 M sodium bicarbonate (pH 8.3). The mixture was left to incubate in the dark for 15 min at room temperature. Zeba 7K MWCO columns (Thermo Scientific) were equilibrated by adding 1 ml 10 mM phosphate buffer (pH 7.4) and centrifuging at 1000***g*** for 2 min at 4 °C. The eluate was discarded and the process was repeated three times. The protein/dye mixture was added drop-wise onto the top of the column immediately followed by 40 μl phosphate buffer and was centrifuged at 1000***g*** for 2 min at 4 °C. The protein solution was kept on ice and the absorbance at 280 nm (*A_280_*) was measured using a NanoDrop spectrophotometer. The protein concentration was calculated using the *A_280_*, and the molar extinction coefficient of dGAE (1400 cm^−1^ M^−1^) taking into account the absorption of the dye at *A_494_*. dGAE has 14 lysine residues, which are all potential sites at which the 488 dye can bind. The extent of labelling was determined by the *A_494_* and the molar extinction coefficient of the dye (71,000 cm^−1^ M^−1^). The protein (dGAE-488) was used immediately for subsequent experiments or subjected to agitation *in vitro*.

### *In vitro* assembly of dGAE and dGAE-488

*In vitro* assembly of dGAE and dGAE-488 was performed as previously described [41] without reducing agent. Briefly, 100 μM protein was diluted in 10 mM phosphate buffer (pH 7.4) and incubated at 37 °C whilst agitating with a speed of 700 rpm on an Eppendorf ThermoMixer® for 72 h. Samples were visualised using negative-stain TEM. All experiments were conducted using the stock 100 μM dGAE in phosphate buffer.

### Negative-stain TEM

Aliquots (4 μl) of dGAE assembly mixtures (100 μM in phosphate buffer (pH 7.4)) were placed on 400-mesh carbon-coated grid and incubated for 1 min. After removing excess solution with filter paper, the grid was washed with 4 μl filtered Milli-Q water for 1 min and blotted. The grids were negatively stained with 4 μl filtered 2% (w/v) uranyl acetate for 1 min, blotted dry and left to air dry for at least 5 min. Grids were examined on a JEOL JEM1400-Plus Transmission Electron Microscope at 100 kV and electron micrograph images were collected using 4k × 4k One View (Gatan) camera.

### Circular dichroism spectroscopy

Circular dichroism (CD) spectroscopy was performed using a Jasco Spectrometer J715 and spectra were collected in triplicate at a maintained temperature of 21 °C. Protein samples were placed into 0.02-mm path-length quartz cuvettes (Hellma) and scanned from 180 to 320 nm. CD data were converted to molar ellipticity (deg·cm^2^·dmol^−1^).

### Cell culture and dGAE treatment

Undifferentiated SH-SY5Y human neuroblastoma cells were grown in Dulbecco's modified Eagle medium/Nutrient Mixture F-12 (DMEM/F-12) supplemented with 10% foetal calf serum, 1% penicillin/streptomycin (P/S) and 1% l-glutamine. For differentiation, SH-SY5Y cells were plated at a density of 50,000 cells per well in 24-well plates or 300,000 cells per well in 6-well plates. For immunofluorescence, cells were plated onto Menzel-Gläser coverslips (Thermo Scientific). For live-cell imaging, cells were plated on 35-mm dishes on a 1.5-mm coverslip (Mattek). On the first day of differentiation, media was replaced with low-serum culture media (DMEM/F-12 containing 1% foetal calf serum, 1% P/S and 1% l-glutamine) containing 10 μM all-*trans* retinoic acid (RA) (Sigma-Aldrich) and cells were incubated for 48 h. On day 3, this process was repeated with fresh RA and cells were incubated for a further 48 h. On day 5, the cells were washed once in serum-free culture media to remove traces of serum. Serum-free culture media (DMEM/F-12 containing 1% P/S and 1% l-glutamine) containing 50 ng/ml brain-derived neurotrophic factor (STEMCELL Technologies) was added to the cells and incubated for 48 h. Differentiated cells (dSH-SY5Y) were ready to use for experiments on day 7. For dGAE treatment, cells were exposed to 1 μM dGAE (either soluble without agitation or after agitation) and incubated for 24 h. All experiments were conducted using dSH-SY5Y cells.

### Cell viability assay

Following the addition of dGAE, cell viability was measured using ReadyProbes® Cell Viability Imaging Kit (Life Technologies). The kit contains a NucBlue® reagent to label all cells (blue) and a NucGreen® reagent to label dead cells only (green). For tagged dGAE, NucRed® reagent was used to label dead cells (red). One drop of each reagent was added to cells in 500 μl media as described in the manufacturers protocol (Life Technologies). Cells were incubated with the reagents at 37 °C for 15 min and the media was replaced with Live Cell Imaging Solution (Invitrogen™, Thermo Scientific). Cells were imaged using a Zeiss Cell Observer Axiovert 200 M microscope. DAPI fluorescence was captured using a G 365 excitation filter and a LP 420 emission filter with a FT 395 dichroic. Green fluorescence was captured using a FITC filter set (BP 450–490 excitation filter, BP 515–565 emission filter and FT 510 dichroic). Identical acquisition settings were used for all replicates, and images were analysed using FIJI. Six fields of view were taken per sample, and an average of 4500 cells were analysed per condition. The proportion of buffer-treated cell death was quantified by converting the images to grayscale followed by manually adjusting the threshold and converting it into a binary image to highlight live DAPI-stained cells. The number of cells was automatically counted. The dead cells were counted in the same way, and cell death was expressed as a percentage .

### Cell lysis and fractionation

Cells were detached from the coverslip by incubation in 0.25% trypsin–EDTA (Gibco™) and then mixed with 5 ml culture media. The cells were harvested by centrifugation at 500***g*** for 5 min, and the supernatant was discarded. The cell pellet was resuspended in ice-cold PBS and centrifuged at 500***g*** at 4 °C. Cells were lysed in 1% Triton lysis buffer (1% Triton X-100 (v/v), 150 mM NaCl and 50 mM Tris–HCl (pH 7.6)) containing Halt™ protease inhibitors (Thermo Scientific) and phosphatase inhibitors (Thermo Scientific) for 15 min on ice. The samples were centrifuged at 16,000***g*** for 30 min at 4 °C, and the supernatant was collected (Triton-soluble fraction). The pellet was suspended in SDS lysis buffer (1% SDS (w/v), 150 mM NaCl and 50 mM Tris–HCl (pH 7.6)) containing protease and phosphatase inhibitors. The samples were centrifuged at 16,000***g*** for 30 min at room temperature, and the supernatant was collected (Triton-insoluble fraction). Protein concentration in the Triton-soluble fractions was determined using the Pierce™ BCA Protein Assay Kit (Thermo Scientific) according to the manufacturer's instructions.

### SDS-PAGE and Western blotting

For cell lysates, Triton-soluble protein (20 μg) and an equal volume of Triton-insoluble protein were each mixed with Læmmli sample buffer (4 ×) (Bio-Rad Laboratories) containing 5% (v/v) β-mercaptoethanol and heated at 95 °C for 5 min. For recombinant protein, 3 μg was mixed with sample buffer without reducing agent or boiling. All samples were centrifuged for 5 min at 1000***g***, and samples were loaded onto 4–20% Mini-PROTEAN® precast gels (Bio-Rad Laboratories) and run at 120 V for 1 h in Tris-glycine running buffer (25 mM Tris, 192 mM glycine (pH 8.3)), or until the sample buffer reached the end of the gel. For Coomassie staining, the gel was washed three times in double distilled water for 5 min and stained with Imperial™ protein stain (Thermo Scientific) for 1 h and then destained overnight in double distilled water. The stained gel was scanned using an HP Photosmart C5280 scanner. For western blotting, the separated proteins on the gel were transferred to nitrocellulose membrane (0.45 μm) at 200 mA for 90 min. The membranes were blocked in 5% (w/v) bovine serum albumin (BSA) in Tris-buffered saline (50 mM Tris–HCl (pH 7.4), 150 mM NaCl) containing 0.1% (v/v) Tween-20 (TBS-T) for 1 h rocking at room temperature. Membranes were incubated with primary antibodies diluted in 5% BSA in TBS-T overnight at 4 °C. The following primary antibodies and dilutions were used: Anti-Tau (polyclonal, total tau) (Thermo Scientific) (1:2500), AT180 (anti-pT231) (Thermo Scientific) (1:1000), AT8 (anti-pS202-T205) (1:1000) (Thermo Scientific), and anti-GAPDH (1:5000) (Abcam). The next day, membranes were incubated in horseradish peroxidase–anti-mouse antibody (1:5000) (Sigma-Aldrich) or horseradish peroxidase–anti-rabbit antibody (1:5000) (Abcam) in 5% BSA w/v in TBS-T for 1 h at room temperature. The membranes were washed 3 × 10 min in TBS-T between antibody incubations. Immunoreactive protein bands were detected using ECL substrate (Clarity™, Bio-Rad), and X-ray films were scanned using an HP Photosmart C5280 scanner. FIJI was used to quantify the bands in arbitrary densitometry units. The density of bands corresponding to full-length tau (50–70 kDa) was determined and normalised against amount of GAPDH. These values were used to calculate the proportion of total tau that is phosphorylated, expressing the proportions as a percentage of the buffer-treated control group. For the quantification of Triton-insoluble and soluble protein, the proportion of tau in the insoluble and soluble fraction was calculated using the equations: *insoluble/(soluble + insoluble)* or *soluble*/(*soluble + insoluble*) respectively, and expressed as a percentage of the respective soluble or insoluble control value.

### Immunofluorescence

Cell culture medium was aspirated from dSH-SY5Y cells and washed once with PBS. Cells were fixed in 4% (w/v) paraformaldehyde (PFA) in PBS for 15 min followed by three washes in PBS. For permeabilization, the cells were incubated in 0.25% (v/v) Triton X-100 in PBS for 10 min. Cells were blocked in 2% (w/v) BSA in PBS for 1 h. Primary antibodies diluted in 2% (w/v) BSA in PBS and were incubated with the cells for 1 h. The primary antibodies and dilutions used were as follows: AT180 (anti-pT231) (1:250) (Thermo Scientific), AT8 (anti-pS202-T205) (1:500) (Thermo Scientific) and Tau1 (dephosphorylated tau at S195, 198, 199 and 202) (Merck Millipore) [43]. Cells were incubated with goat anti-mouse-Alexa Fluor® 594 (1:1000) (Invitrogen™, Thermo Scientific) diluted in 2% (w/v) BSA in PBS for 1 h in the dark. Cells were washed three times in PBS between antibody incubations. Cells were mounted onto glass slides using Prolong^TM^ Gold Antifade Moutant with DAPI (Thermoscientific) mounting medium containing 4,6-diamidino-2-phenylindole (DAPI). Mounted slides were stored in the dark at room temperature for 24–48 h before imaging and kept at 4 °C for long-term storage. Cells were imaged using Leica SP8 confocal microscope.

### Labelling of acidic organelles

Cells were plated on 35-mm dishes on a 1.5-mm coverslip (Mattek). Following differentiation, dGAE-488 (5 μM) was added to the cells for 24 h. For labelling lysosomes and endosomes, LysoTracker® red (Life Technologies) was diluted in culture media at a final concentration of 50 nM and incubated for 90 min before imaging using a Leica SP8 confocal microscope.

### Confocal microscopy

All confocal microscopy imaging was carried using a Leica SP8 confocal microscope. The instrument setting used PMT 3 and PMT Trans channels/lasers and images were acquired with a HC PLAPoCs2 63 x/1.40 oil-immersion objective lens. Samples were scanned sequentially to prevent spectral bleed through. All images were collected as Z-stacks for all channels using a step size of 0.5 μm. Five to ten Z-stacks were taken for each sample, and each experiment was repeated three times or more. To monitor live uptake of dGAE-488, the environment was maintained at 37 °C with humidified CO_2_ and the Adaptive Focus Control feature was used to maintain constant focal planes throughout the course of the experiment.

### Processing cells for TEM

dSH-SY5Y cells were treated with Alexa Fluor® 488 containing buffer, or 10 μM freshly prepared dGAE-488 for 24 h. The cells were washed once in PBS, scraped into a tube and centrifuged at 500***g*** for 5 min. The media was removed and the cells were suspended in a 1:1 mixture of pre-warmed culture media: 4% (v/v) PFA for 15 min at 37 °C. The cells were centrifuged at 500***g*** for 5 min  and the cells were resuspended in fresh 4% (v/v) PFA and 0.1% (v/v) glutaraldehyde (GA) in 0.1 M phosphate buffer (pH 7.4) for 3 h at room temperature. The fixed cells were centrifuged at 1000***g*** for 5 min. The supernatant was discarded, and the pellet was suspended in 50 mM glycine in PBS for 10 min at room temperature. The cells were centrifuged at 1000***g*** for 5 min, and the pellet was washed three times with 0.1 M cacodylate buffer (pH 7.4). Around 200 μl 4% (w/v) low melting point agarose was added to the cells and immediately centrifuged at 1000***g*** for 10 min at 30 °C. The tube was immediately transferred to 4 °C or on ice for 20 min to solidify the agarose. The agarose-embedded cell pellet was transferred to a new tube and washed 2–3 times with 0.1 M cacodylate buffer (pH 7.4). The pellet was post-fixed in a reduced osmium solution (1% (v/v) osmium tetroxide, 1.5% (w/v) potassium ferrocyanide in 0.1 M cacodylate buffer, pH 7.4) for 1 h at 4 °C followed by washing three times in 0.1 M cacodylate buffer (pH 7.4) and three times in double-distilled H_2_O for 5 min each. The pellet was dehydrated in an ethanol series consisting of 30%, 50%, 75%, 90% and 95% ethanol for 15 min each at 4 °C followed by three incubations in 100% ethanol for 20 min each at 4 °C. The sample was then infiltrated in a 2:1 mixture of 100% ethanol:Unicryl™ resin (for 30 min followed by a 1:2 mixture of 100% ethanol:Unicryl™ resin (BBI Solutions) for 30 min. Finally, the pellet was transferred to a BEEM capsule (Agar Scientific) and infiltrated in complete Unicryl™ resin overnight at 4 °C. The resin was cured using light polymerisation for 48 h by illumination from the underside of the BEEM capsules from a 12 V, 100 W type 6834 Philips projection lamp at a distance of 35 cm. Ultrathin sections (70 nm) were taken using a diamond knife on a Leica EM UC7 ultramicrotome fitted with a Leica M80 microscope (Leica Microsystems) and placed on 300 Mesh Hexagon Nickel 3.05-mm grids (Agar Scientific Ltd) before proceeding with immunogold labelling.

### Immunogold labelling TEM

A modified PBS (pH 8.2) containing 1% BSA, 500 μl/L Tween-20, 10 mM Na-EDTA and 0.2 g/L NaN_3_ (termed PBS +) was used throughout the following procedures for all dilutions of antibodies and gold probes. The ultrathin sections were initially blocked using normal goat serum (diluted 1:10 dilution in PBS +; Sigma-Aldrich) for 30 min at room temperature and then incubated with anti-Alexa Fluor® 488 primary antibody (1:50) (Thermo Scientific). The sections were washed three times in PBS + for 2 min each followed by incubation with 10-nm gold particle-conjugated goat anti-rabbit IgG secondary probe at a 1:10 dilution in PBS + for 1 h at room temperature. The sections were washed three times in PBS + for 10 min each and four times in distilled water for 5 min each. Immunogold-labelled thin sections were subsequently post-stained in 2% (w/v) uranyl acetate for 1 h before imaging on the TEM.

### Image analysis

FIJI (https://fiji.sc) imaging processing package [44] was used for all image analysis. For quantification of fluorescence intensities, images were Z-projected to maximal intensity. Five to ten fields of view were taken from each condition, and an average of 50 cells per condition were subjected to analysis. Firstly, a region of interest (the cell body) was drawn around an individual cell, excluding cells that had fused nuclei. Area-integrated intensity and mean grey value were measured as well as three selections from around the cell with no fluorescence (background). The corrected total cell fluorescence (CTCF) was then calculated as CTCF = integrated density − (area of selected cell × mean fluorescence of background readings.

For quantification of internalised dGAE-488, a focal plane from the middle of the cell that contained maximal DAPI fluorescence was selected and subjected to analysis.

### Data analysis

Data and statistical analyses were performed using Microsoft Excel and GraphPad Prism 7. All data are expressed as the mean ± SEM. When comparing two groups, Student's unpaired t-test with Welch's correction was used to determine statistical significance. When comparing more than two groups, one-way ANOVA with Dunnet's post-hoc test was used to determine differences between experimental groups and a control group. Differences were considered to be statistically significant if *p* < .05.

## Results

### dGAE and dGAE-488 form structurally and morphologically similar fibrils

**Figure 1:**
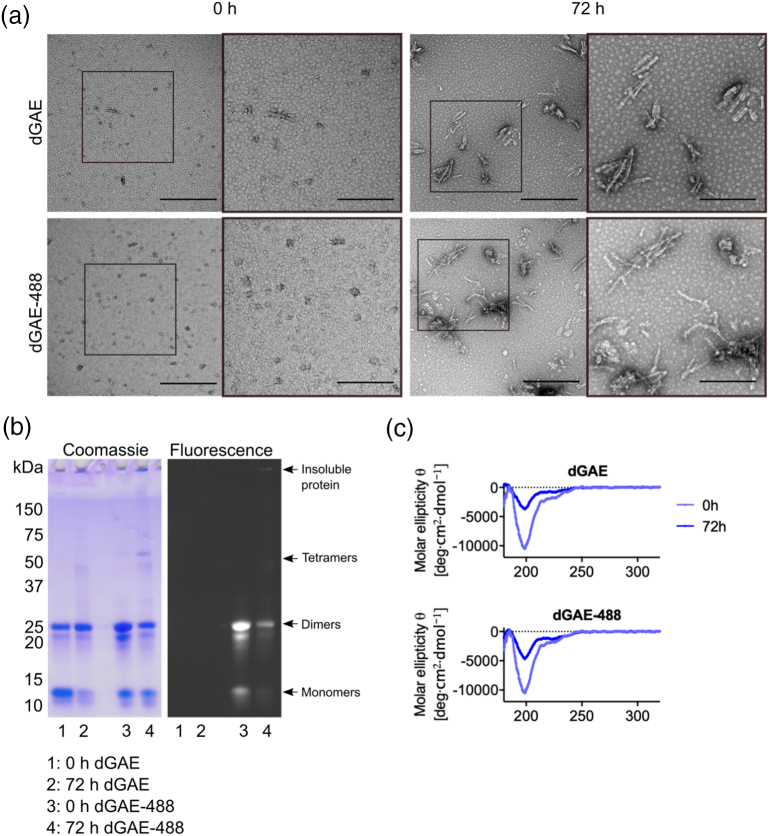
dGAE and dGAE-488 self-assemble to form structurally similar fibrils. dGAE and dGAE-488 were incubated at 100 μM for 72 h. Aliquots of assembly mixture were taken before (0 h) and after (72 h) fibrillisation for negative-stain TEM, SDS-PAGE gel electrophoresis and CD spectroscopy. (a) Electron micrographs of dGAE species at 0- and 72-h agitation. The scale bar represents 500 nm. Middle panel is a higher magnification of the white box in the left panel (scale bar, 200 nm). (b) Non-reducin24g SDS-PAGE gel of dGAE and dGAE-488 shown as Coomassie stain (left panel) and fluorescence (right panel). Black arrowheads point to monomers and dimers, tetramers and insoluble fibrils in the well. (c) CD spectra of the whole assembly mixture for dGAE and dGAE-488 at 0- and 72-h agitation.

Soluble dGAE was fluorescently labelled using an Alexa-fluor 488® tag (dGAE-488) to enable internalisation of dGAE assemblies to be monitored and to distinguish exogenous dGAE from endogenously expressed tau. On average, there were 0.1 mol of 488 dye per mole of protein after every labelling reaction. The Alexafluor-tag® labels amino groups at the N terminus and on lysine residues, and hence, we examined the tagged and untagged dGAE using TEM, SDS-PAGE and CD spectroscopy to determine whether the label affects aggregation and/or filament formation. Using the non-reducing conditions for aggregation established previously [41], dGAE and dGAE-488 produced morphologically similar short twisted fibrils (Figure 1(a)). SDS-PAGE of dGAE and dGAE-488 showed the presence of both the 10/20-kDa and of the 12/24-kDa forms (monomer/dimer), with the latter predominating in the non-agitated dGAE preparation at 0 h. We have previously shown that dGAE is random coil at 0 h and consists mainly of SDS soluble monomer and dimer, although the solution likely contains a mixture of low molecular weight species. Over time, the dGAE self-assembles to form β-sheet rich filaments [41]. The dGAE-488 preparation showed slightly increased intensity of dimer bands at 0 h. Electron micrographs of the proteins at 0 and 72 h show similar size species in both the dGAE and dGAE-488 preparations (Figure S1), with round species at 0 h ranging from 10 to 80 nm in diameter (Figure S1Aii) and fibrils at 72 h ranging from 20 to 350 nm in length (Figure S1Bii). After fibrillisation induced by agitation for 72 h, there was less of the 12-kDa monomeric form of dGAE compared with dGAE-488, and more of both preparations were retained in the gel well [41]. CD spectra were similar for both preparations, with similar intensity minima at 198 nm (predominantly random coil conformation) at 0 h and the expected decrease in random coil signal by 72 h, which accompanied an increase in insoluble β-sheet structures with a minimum around 218 nm. We have previously demonstrated the β-sheet signal at 218 nm in the pellet following centrifugation to separate it from supernatant [41].

### Extracellularly applied aggregated dGAE but not soluble dGAE induces acute cell death

**Figure 2:**
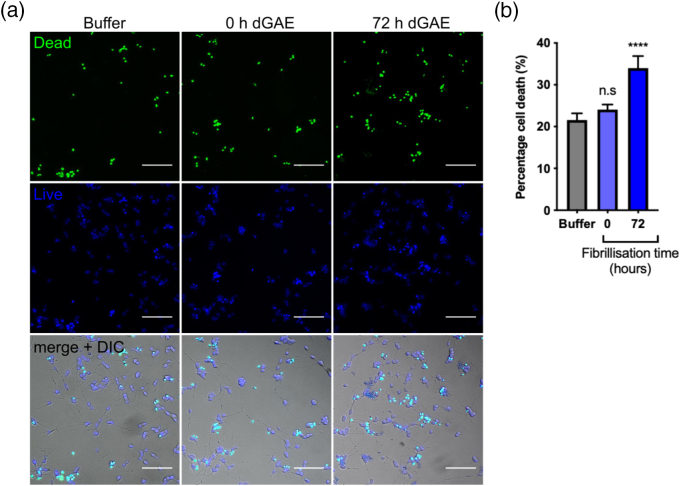
Exposure to aggregated dGAE but not soluble (monomer/dimer) dGAE results in increased cell death. dGAE (100 μM) was agitated for 72 h to produce fibrils. Soluble (0 h) or aggregated (72 h) species (1 μM) were added to cells and left to incubate for 24 h. (a) Representative wide-field images following exposure to buffer or dGAE with ReadyProbes® reagent, showing total nuclei in blue and nuclei of dead cells in green. The scale bar represents 100 μm. (b) The percentage cell death was quantified for all conditions. Data are presented as mean values from six fields of view from four to six independent experiments ± SEM. A one-way ANOVA shows a significant difference between groups (*F* = 11.26, *R^2^* = 0.12, *p* < .0001). Dunnett's multiple comparisons show a significant difference between cells treated with buffer only (21.6% ± 1.6%) and cells treated with dGAE fibrils (72 h dGAE) (34.0% ± 2.9%) (*p* < .0001) but not between buffer-treated cells and soluble dGAE-treated cells (24.1% ± 1.2%).

Aggregated dGAE (100 μM agitated for 72 h) and soluble dGAE (100 μM 0 h, no agitation) were applied at a concentration of 1 μM directly to dSH-SY5Y cells and incubated for 24 h. Cell viability was measured after 24 h using the ReadyProbes® assay to measure cell death (Figure 2(a)). Although there was some increase in the percentage of dead cells due to soluble dGAE compared with buffer treatment, this did not reach statistical significance (Figure 2(b)). There was a significant increase in cell death following incubation with aggregated dGAE (34 ± 2.9%, *p* < .0001) compared with buffer only control (21.6% ± 1.6%). Increasing the concentration of soluble dGAE up to 20 μM produced no further increase in cell death after incubation for 24 h (figure S2). dGAE and dGAE-488 showed comparable effects on cell death following the incubation of cells with 1 μM soluble or aggregated protein, although the soluble form of dGAE-488 was marginally more toxic, but with no difference after 72 h fibrillisation (figure S3).

### Labelled dGAE-488 is internalised by dSH-SY5Y cells

**Figure 3:**
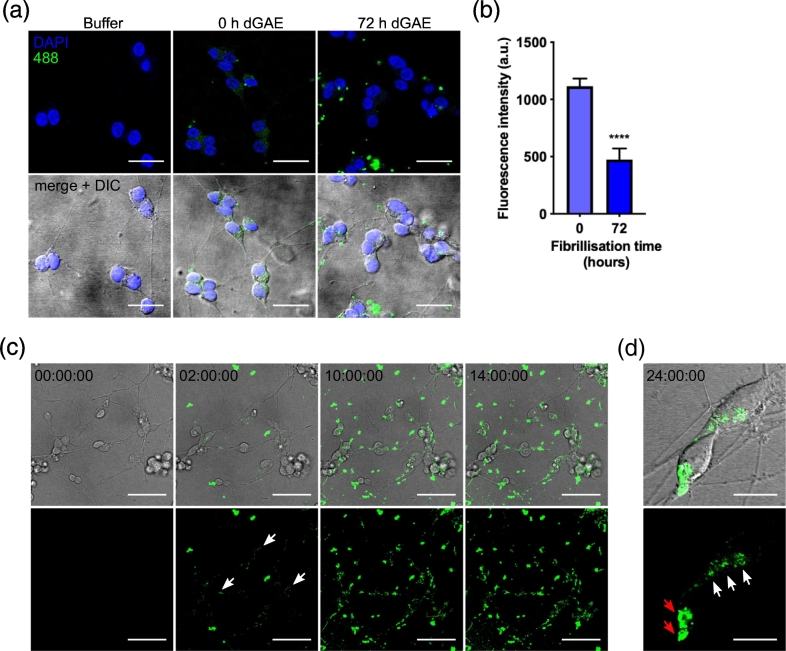
Soluble dGAE-488 readily internalises into dSH-SY5Y cells. (a) 1 μM soluble dGAE-488 (unagitated) or 72-h agitated (aggregated) dGAE was added to the media of dSH-SY5Y cells and then fixed and visualised by confocal microscopy after 24-h exposure. All scale bars represent 20 μm. (b) The 488 fluorescence intensity in the cell body was quantified from the middle of the z-stack from N = 273 cells from six independent experiments (0 h) and from N = 120 cells (72 h) from three independent experiments. An unpaired *t*-test with Welch's correction shows a significant difference in 488 fluorescence intensity between 0 h (1114 ± 66.75 AU) and 72 h (474.1 ± 95.97 AU) (*t* = 5.475, *df* = 237.7, *R^2^* = 0.112, *p* < .0001). Data are shown as mean ± SEM. (c) Internalisation of 5 μM soluble dGAE-488 (unagitated) was monitored live using confocal microscopy from *t* = 0 h to *t* = 14 h. 488-positive puncta can be seen internalised into the cell body and neurites from 2 h following the initial addition of dGAE-488 (white arrows). The scale bar represents 50 μm. (D) Higher magnification images of internalised soluble dGAE-488 after 24-h exposure show a punctate pattern in the cell body (white arrow) and larger perinuclear accumulation (red arrow). The scale bar represents 15 μm. One z-slice is shown from the middle of the cell body for all panels.

Next, we investigated the uptake of soluble and aggregated dGAE into dSH-SY5Y cells and examined whether aggregation state affects the efficiency of internalisation. We used the labelled dGAE-488 form to permit uptake to be visualised and measured having corrected for total cell fluorescence. Soluble or agitated (72 h) dGAE-488 was incubated with dSH-SY5Y cells at 1 μM for 24 h. Confocal microscopy analysis showed that following exposure to soluble dGAE-488, fluorescence was observed within the cells as punctate staining (Figure 3(a)). Aggregated dGAE-488 was internalised significantly less efficiently than the soluble form (474 ± 96 *versus* 1114 ± 67 AU per cell, *p* < .0001; Figure 3(b)), and larger accumulations of dGAE-488 fluorescence could be observed outside the cells. Live cell imaging was used to monitor the internalisation of soluble dGAE-488. Some dGAE-488 remains outside cells and increases in brightness and size over time, consistent with some continued assembly in the medium. Internalised dGAE-488 could be detected within neurons after 2 h of incubation and uptake was found to increase over time (Figure 3(c)). By 24 h, both distinct punctate staining pattern and larger perinuclear accumulations were observed (Figure 3(c) and (d)). Although the time intervals of the live-cell imaging did not allow for detailed examination of intracellular transport, the internalised dGAE-488 was observed being trafficked along the processes (Figure S4 movie).

### Internalised dGAE-488 leads to increased phosphorylation of endogenous tau

**Figure 4:**
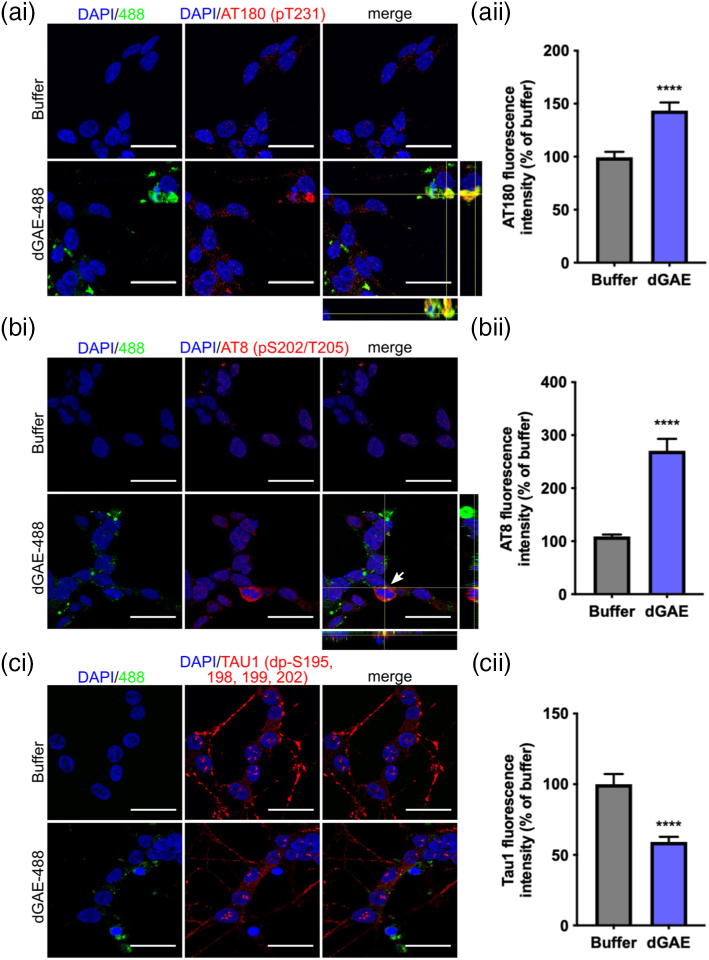
Exposure to dGAE-488 leads to an accumulation of endogenous phospho-tau in dSH-SY5Y cells. Soluble dGAE-488 of 1 μM (unagitated) was added to the media of dSH-SY5Y cells for 24 h. Cells were immunolabelled for endogenous tau using p-tau antibodies (ai) AT180, (bi) AT8 and (ci) dephosphorylated Tau-1. One z-slice is shown from the middle of the cell body. Orthogonal views are displayed in ai and bi to show potential colocalisation of dGAE-488 with endogenous tau. The scale bar represents 20 μm. Each panel shows quantification of fluorescence intensity as a percentage of buffer-treated cells. (aii) Quantification of AT180 fluorescence (N = 69 cells (buffer), 76 cells (dGAE) from four independent experiments). An unpaired *t*-test with Welch's correction shows a significant difference in AT180 fluorescence intensity between dGAE- (143.5% ± 7.8%) and buffer-treated cells (100% ± 5.2%) (*t* = 4.702, *df* = 128.2, *R^2^* = 0.1471, *p* < .0001). (bii) Quantification of AT8 fluorescence. N = 348 cells (buffer), N = 338 cells (dGAE) from three independent experiments. An unpaired *t*-test with Welch's correction shows a significant difference in AT8 fluorescence intensity between dGAE- (270.7% ± 22.5%) and buffer-treated cells (100% ± 3.51%) (*t* = 7.103, *df* = 353.4, *R^2^* = 0.1249, *p* < .0001). (cii) Quantification of Tau1 fluorescence. N = 68 cells (buffer), 96 cells (dGAE) from three independent experiments. An unpaired *t*-test with Welch's correction shows a significant difference in Tau-1 fluorescence intensity between dGAE (59.17% ± 3.6%) and buffer-treated cells (100% ± 7.3%) (*t* = 5.011, *df* = 99.84, *R^2^* = 0.201, *p* < .0001).

Our results suggest that incubation with aggregated dGAE leads to significant cell death after incubation for only 24 h, whereas soluble dGAE is internalised but does not appear to be significantly toxic following a 24-h incubation. Therefore, we investigated whether internalised soluble dGAE-488 may be capable of altering the aggregation or phosphorylation state of endogenous tau. Following exposure to soluble 1 μM dGAE-488, cells were fixed and immunolabelled using antibodies recognising tau epitopes outside of the dGAE sequence allowing specific detection of only endogenous tau (Figure 4(a)–(c)). Cells were examined for colocalisation of internalised dGAE-488 and endogenous tau, and the fluorescence intensity of labelled tau in the cell body was quantified in individual cells for each antibody. Labelling with AT180 (tau phosphorylated at T231; pT231) and AT8 (tau phosphorylated at 202–205; pS202–pT205) was largely absent in buffer-treated cells, but increased significantly following incubation with soluble dGAE-488, with some cells showing colocalisation of dGAE-488 with phosphorylated endogenous tau (Figure 4(a) and (b)). We have previously shown that tau dephosphorylated between amino acids 192–204 detected, using Tau-1 antibody, is found in the nucleus in dSH-SY5Y cells [43]. Here, we identified tau-1 labelling in the nucleus as expected but found that there was no colocalisation with dGAE-488. Quantification of Tau-1 fluorescence showed a significant decrease in fluorescence intensity in cells treated with dGAE-488 (Figure 4(c)), which may indicate that the tau becomes phosphorylated.

### Exposure of cells to soluble dGAE-488 leads to an increase in triton-insoluble endogenous tau

**Figure 5:**
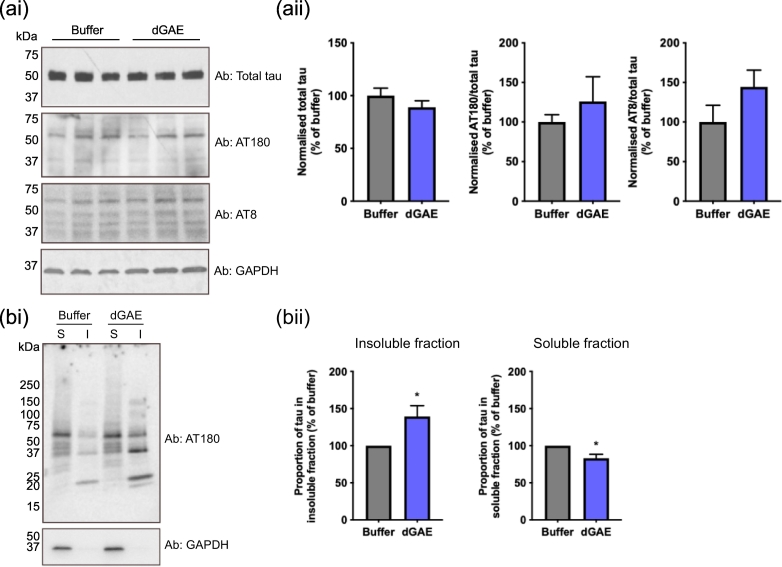
Exposure to dGAE for 24 h leads to an accumulation of endogenous phospho-tau in the Triton-insoluble fraction of dSH-SY5Y cells. (ai) Representative Western blot of SH-SY5Y cell lysate. Soluble dGAE of 1 μM (unagitated) was added to the media of dSH-SY5Y cells. After 24 h, cells were lysed in RIPA buffer and run on SDS-PAGE. Blots were probed against total tau, AT180 and AT8. GAPDH was used as a loading control. (aii) The intensity of bands at 50–70 kDa were quantified for each antibody and normalised against GAPDH. The normalised values for AT180 and AT8 are expressed as a proportion of total tau (percentage of buffer). Data are shown as mean ± SEM from three independent experiments. An unpaired *t*-test shows no significant difference in total tau (*p* = .3101), AT180 (*p* = .4662) or AT8 (*p* = .2119) immunoreactivity between buffer-treated control and dGAE-treated cells. (bi) Cells were also sequentially lysed in Triton-X 100 lysis buffer and run on SDS-PAGE. S = Triton-X 100 soluble lysate; I = Triton-X 100 insoluble lysate. The AT180-antibody was used to detect endogenous phosphorylated tau and GAPDH was used as a loading control. (b) The proportion of tau in the insoluble and soluble fractions was quantified by densitometry and expressed as a percentage of buffer-treated controls. Data are shown as mean ± SEM from four independent experiments (one biological repeat per experiment). An unpaired *t*-test shows a significant difference in insoluble tau between buffer- (100 ± 0) and dGAE-treated cells (139.4 ± 14.54) (*t* = 2.709, *df* = 6, *R^2^* = 0.5501, *p* = .0352) and in soluble tau between buffer (100 ± 0) and dGAE-treated cells (82.98 ± 5.48) (*t* = 3.106, *df* = 6, *R^2^* = 0.6165, *p* = .0201).

Western blots of the whole cell lysates were performed to examine the effect of exogenously applied dGAE on endogenous tau phospho-epitopes. Comparison of cells incubated with soluble dGAE with buffer-treated control cells revealed no change in levels of total tau and marginal increases in AT8 or AT180 immunoreactivity that did not reach statistical significance. Whilst western blots measure total levels of phospho-tau in cells, immunofluorescent imaging is able to highlight subcellular localisation of any increases. In order to determine the effects of internalised soluble dGAE-488 on the solubility of endogenous tau in neurons, we undertook sequential extraction of tau protein from cell lysates following solubilisation with 1% Triton X-100 and centrifugation (16,000***g*** for 30 min). Following incubation with soluble dGAE for 24 h, there was a clear redistribution of tau protein immunolabelled with AT180 from Triton-X 100-soluble to Triton-X 100-insoluble fractions following incubation with soluble dGAE compared with buffer-treated controls. dGAE incubation produced a reduction in soluble tau immunoreactive with AT180 (83.0% ± 5.5%, *p* = .0201) and an increase in the insoluble form (139.4% ± 14.5%, *p* = .0352) (Figure 5(a) and (b)). Unexpectedly, a small amount of tau was observed in the insoluble fraction of the buffer-treated control, but the difference between control and treated cells was significant. Furthermore, a new AT8-positive truncated tau species having gel mobility of 20–25 kDa appeared in the insoluble fraction. This was more intense in the fraction from cells incubated with dGAE than with buffer alone.

### Internalised dGAE-488 is localised to perinuclear acidic vesicles

**Figure 6:**
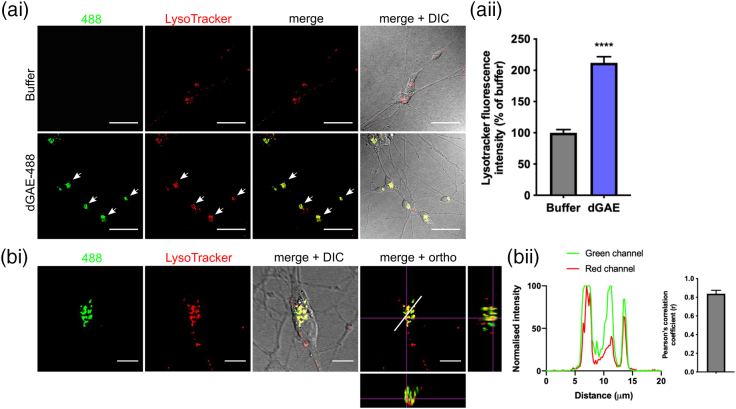
Internalised soluble dGAE-488 is localised to acidic vesicles in dSH-SY5Y cells. (ai) Representative immunofluorescence images of cells exposed to 1 μM soluble dGAE-488 (unagitated) for 24 h. Cells were labelled with LysoTracker® to stain for acidic vesicles including lysosomes and endosomes, and were imaged live. dGAE-488 and LysoTracker® labelling is mainly localised in the cell body (white arrows). One z-slice is shown from the middle of the cell body. The scale bar represents 50 μm. (aii) Quantification of LysoTracker® fluorescence intensity as a percentage of buffer-treated cells. Data show mean ± SEM pooled from three independent experiments. N = 170 cells (buffer), N = 146 cells (dGAE). An unpaired *t*-test with Welch's correction shows a significant difference in LysoTracker® fluorescence intensity between dGAE (211.9% ± 9.89%) and buffer-treated cells (100% ± 5.23%) (*t* = 10, *df* = 222.6, *R^2^* = 0.3101, *p* < .0001). (bi) Higher magnification of a single cell labelled with LysoTracker® containing internalised dGAE-488. The last panel displays orthogonal views to show colocalisation of dGAE-488 with LysoTracker®. One z-slice is shown from the middle of the cell body. The scale bar represents 10 μm. (bii) Normalised values of fluorescence intensity for 488 (green) and LysoTracker (red) along the region indicated by the white line (20 μm) in the last panel in (bi). Colocalisation of dGAE-488 and LysoTracker® is confirmed by the Pearson's correlation coefficient between the green and red channel. The average Pearson's *R* value was 0.8382 ± 0.036 (*p* < .0001 for each cell) (N = 7 cells).

Intracellular fluorescent punctate particles were observed within the cytoplasm, close to the nucleus after 24 h of exposure of neurons to soluble dGAE-488. This staining pattern was consistent with its localisation in cytosolic vesicles. We therefore examined whether internalised dGAE-488 was localised to endosomal/lysosomal compartments. Soluble dGAE-488 was incubated with cells for 24 h and stained with LysoTracker®, a dye that labels acidic organelles (lysosomes and late endosomes), for 30 min and then fixed and visualised by confocal microscopy. There was a significant increase in the intensity of the LysoTracker® fluorescence in cells exposed to soluble dGAE-488 compared to buffer-treated control cells (211.9% ± 9.9%, *p* < .0001; Figure 6(ai) and (aii)). Internalised dGAE-488 showed a strong colocalisation with LysoTracker® following quantification in Z-stacks using the Pearson's correlation coefficient (0.8382 ± 0.036, *p* < .0001; Figure 6(bi) and (bii)).

### Internalised dGAE-488 is packaged in vesicular compartments

**Figure 7:**
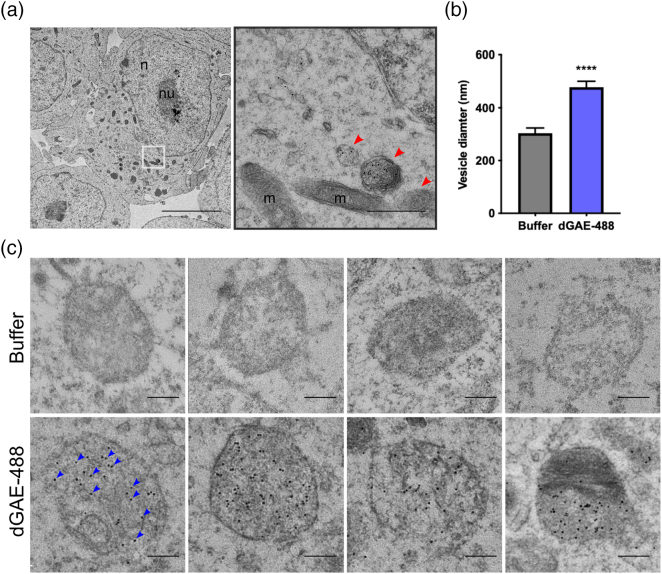
Internalised soluble dGAE-488 is localised to vesicular structures in dSH-SY5Y cells. dSH-SY5Y cells were exposed to 1 μM soluble dGAE-488 (unagitated) for 24 h and were processed for immunogold electron microscopy. Anti-488 antibody was used to label dGAE, detected by 10-nm gold particles. (a) Electron micrograph showing preserved ultrastructure of cells, with the main cellular compartments labelled: n, nucleus; nu, nucleolus; m, mitochondria; v, vesicular structure. Red arrowheads point to vesicular structures. The black outlined image represents a magnified image of the area in the white box. Left: the scale bar represents 5 μm. Right: the scale bar represents 1 μm*.* (b) Quantification of vesicle diameter. Data are shown as mean ± SEM pooled from two independent experiments. N = 34 vesicles (buffer), N = 45 vesicles (dGAE-488). An unpaired *t*-test showed a significant difference in vesicle diameter between buffer (303.7 ± 20.03) and dGAE-treated cells (478.4 ± 22.48) (*t* = 5.601, *df* = 77, *p* < .0001, *R^2^* = 0.2895). (c) Higher magnification electron micrographs of vesicular structures from buffer-treated cells and dGAE-488 treated cells. Blue arrowheads point to some examples of gold particles. The scale bar represents 200 nm.

TEM was used to examine the ultrastructure of accumulated tau and dGAE-488 in cells. dSH-SY5Y were exposed to 10 μM soluble dGAE-488 for 24 h and were processed for immunogold TEM using an antibody against Alexa Fluor® 488 to label dGAE-488 specifically. Vesicular structures and mitochondria were observed within the sectioned cells confirming that the TEM protocol preserved the cellular structure (Figure 7(a)). Although no clear fibrillar structures labelled with anti-488 in the cytoplasm, examination at higher magnifications revealed the presence of particles densely labelled with anti-488 within membrane-bound vesicular structures suggesting an accumulation of 488-labelled protein in these organelles (Figure 7(c)). Similar vesicular structures were also present in vehicle-treated cells but lacked gold labelling. Analysis to compare diameters of the membrane-bound compartments that showed neurons treated with dGAE-488 cells contained vesicles of larger diameter than buffer-treated control cells (448.4 ± 22.5 nm *versus* 303.7 ± 20.0 nm, *p* < .0001), with the largest vesicles being 700–800 nm and smallest being 200–300 nm (Figure 7(b)).

## Discussion

Previous studies investigating the properties of exogenously applied tau in tissue culture cell models have been hampered by the use of non-neuronal or neuronal *in vitro* models that overexpress human wild-type or mutant tau, often using non-physiological fragments or preparations of tau to initiate aggregation [19,24,25]. We have previously reported morphological similarities between filaments formed *in vitro* from dGAE and PHFs found in AD brain tissue [41,42]. Aggregation of dGAE does not require exogenous seeding factors, and it is possible to control its fibrillisation *in vitro* [41]. This has permitted us to develop a new model system to investigate the internalisation, cytotoxicity and effect on endogenous tau of the PHF-forming region of tau in human neuronal cells in the absence of mutant tau overexpression or exogenous seeding factors.

In the time frame of these experiments (24 h), we observed an increase in cell death following exposure of differentiated neuroblastoma cells to aggregated dGAE but minimal differences in the viability of cells treated with soluble dGAE. Previous studies have reported that full-length tau, mutated tau and tau aggregates with different phosphorylation states have minimal effects on cell viability [[45], [46], [47]]. For instance, it was observed that oligomers formed from a variant repeat tau fragment (corresponding to tau244–372 with a Lys-280 deletion; Tau^RDΔK^) are selectively toxic to dendritic spines without affecting cell viability [47]. In contrast, other studies have shown that toxicity is dependent on the precise fragments of tau that are used [15,[48], [49], [50]]. Variation in tau toxicity assays may be related in part to the differences in tau fragments used and in their methods of tau preparation. Under conditions used here, aggregated dGAE appeared to be more acutely toxic to differentiated neuroblastoma cells following the 24-h incubation period.

Although minimally toxic even at higher concentrations, the dGAE in its soluble form was nevertheless internalised into differentiated neuroblastoma cells within 2-h exposure. This is consistent with previous studies using fluorescently labelled K18 (tau244–372) [28,51] and monomers of full-length tau carrying the pathogenic P301L mutation [46]. After agitation, the dGAE-488 is internalised much less efficiently. This is consistent with another report indicating that heparin-induced tau fibrils and tau filaments extracted from transgenic mice are not readily taken up by cells [26]. In our study, we observed some internalisation using aggregated preparations but this may be due to soluble or partially soluble species that remain in solution during preparation of filaments. It is likely that the agitated dGAE-488 contains a mixture of sizes of tau assemblies, some of which are internalised and some of which are not. Whilst the exact species being internalised remains to be resolved, the soluble dGAE preparation consists of aggregates ranging in diameter from 10 to 80 nm implying a manifold of oligomeric forms (Figure 1(a)). Ongoing studies seek to address this question by separating low molecular weight, oligomeric and fibrillar species. The inherent assembly-competence of dGAE may contribute both to its internalisation and seeding activity [41]. Furthermore, the time taken for 0 h dGAE to become toxic may be longer than the time frame of these experiments. Extracellular tau has been shown to bind to exogenous heparan sulphate proteoglycans and to enter cells by micropinocytosis in human embryonic kidney (HEK) cells and primary rodent neurons [23,26]. On the other hand, two potential uptake mechanisms for monomeric tau (wild-type and P301S) into human stem-cell derived neurons have been reported: a rapid endocytic phase, and a slower micropinocytosis phase [51]. Heparin-induced aggregated tau (P301S) entry was shown to be largely dependent on endocytosis. The internalisation we have described does not require exogenous factors either for aggregation or for cellular uptake.

We have investigated whether endogenous tau could be recruited and converted into insoluble forms following uptake of soluble dGAE into the cytoplasm. The interaction of between endocytosed tau with cytosolic tau has been investigated previously in various cell models that have required the overexpression of human tau [23,25,52], or by introducing exogenous tau with the help of protein delivery reagents [24]. The data presented here show that incubation with soluble forms of dGAE results in a local increase in endogenous phospho-tau in cells alongside normal levels of endogenous tau, consistent with tau pathology in AD. Using dGAE-488 together with antibodies recognising epitopes outside the repeat domain to probe the phosphorylation state of endogenous tau protein, we have shown that there is an increase in phosphorylation of endogenous tau at T231 and S202–T205. These changes are accompanied by an increase in levels of insoluble phosphorylated tau protein after sequential extraction, but not in the whole lysate. Western blots from whole lysate did not reveal any clear differences in phospho-tau levels using AT80 or AT180, which may suggest that overall levels remain constant, but that local phospho-tau levels increase within areas of insoluble/aggregated tau as shown by immunofluorescence and following sequential extraction. The mechanism of induction of aggregation or increased phosphorylation following uptake of soluble forms of dGAE is not known at this stage. This truncated tau species is able to self-assemble spontaneously without any requirement for phosphorylation. Here we show that dGAE co-aggregates with endogenous tau and that these accumulate together within endosomal/lysosomal compartments. Further studies are ongoing to better characterise the nature of the intracellular processing and co-aggregation with endogenous tau that leads to accumulation in the endosomal/lysosomal compartment. One scenario might be that disruption of membranes by dGAE oligomers permit leakage into the cytoplasm and interaction with endogenous tau [15,53,54].

It has been reported previously that internalised tau aggregates colocalise with endosomal and lysosomal markers [26]. Here, we show that the majority of dGAE that is internalised by cells is localised to lysosomal compartments. Using immunogold TEM, we reveal the localisation of dGAE-488 to vesicular structures with a size range suggestive of lysosomal compartments. The immunofluorescence and TEM findings together suggest the accumulation of both dGAE and endogenous tau within the endo-lysosomal compartments. These findings provide a novel perspective on the ultrastructure of cells following internalisation of a pathological form of tau and suggest that the autophagy pathway may be implicated in its degradation, which is consistent with previous studies [26,28,51]. Future studies involving longer incubation times will allow us to examine whether these aggregates can develop further within lysosomal compartments and then in the cytosol. In cell-free and cellular models, the formation of oligomers was required for a template-directed truncation of tau protein and this process [22,39] and their clearance [55] are enhanced by tau aggregation inhibitors.

Physiological and pathological forms of tau are cleared by the proteasomal and autophagic degradative systems [56], and it has been suggested that pathological tau is preferentially degraded by the autophagic-lysosomal system [57,58]. It is possible that the accumulation of pathological tau may interfere with the normal functioning of these degradative processes. In AD, there is support for impaired degradation of autophagic vacuoles by lysosomes [59,60], suggesting that incomplete clearance of pathological tau could contribute to neuronal dysfunction. Understanding how accumulated tau species can be cleared from cells may allow for the development of complementary therapeutic approaches in addition to inhibition of tau aggregation.

In conclusion, the work presented here provides a basis for further research to address unanswered questions concerning the physiological and pathological consequences of PHF-core tau self-assembly. The ease with which soluble dGAE self-assembles and internalises into neuronal cells makes it possible to study the effect of its self-assembly in a cellular environment, in particular its effect on acute toxicity and its influence on phosphorylation state and insolubility of endogenous tau at an ultrastructural level. This approach will provide a useful tool to facilitate the study of tau aggregation in the absence of any overexpression of the protein, post-translational modification, or inducers of assembly and ultimately to study the mechanism of action of tau aggregation inhibitors that target tau seeding and neuronal transmission in AD and related tauopathies.

## Appendix A Supplementary data

Supplementary figures Image 1
