# Supplementary material for: Paired Helical Filament-Forming Region of Tau (297–391) Influences Endogenous Tau Protein and Accumulates in Acidic Compartments in Human Neuronal Cells
Source: J Mol Biol. 2020 Aug 7;432(17):4891–907. doi: 10.1016/j.jmb.2020.05.027 (PMC7427330; doi:10.1016/j.jmb.2020.05.027)
Supplement: Supplementary file 1 — Supplementary figures [file mmc1.docx]

**Paired helical filament-forming region of tau (297-391) core tau unit influences endogenous tau protein and accumulates in acidic compartments in human neuronal cells**

Saskia J Pollack^1^, Jasmine Trigg^1^, Tahmida Khanom,^1^ Karen E Marshall^1^, Youssra Al-Hilaly^1,2^, Janet E Rickard^3^, Charles R Harrington^3,4^, Claude M Wischik^3,4^, Louise C Serpell^1^*

**Supplementary information**

***Figure S1. Size distribution of dGAE species added to cells.*** The size of 0 h and 72 h dGAE and dGAE-488 species were analysed from negative-stain electron micrographs. (Ai) Diameter of 0 h dGAE and dGAE-488 species presented as a scatter column chart of all the values (N = 156 particles analysed for each condition). An unpaired t-test shows no significant difference in diameter of species between 0 h dGAE (25.67 ± 0.821 μm) and 0 h dGAE-488 (25.42 ± 0.573 μm) (t = 0.2528, df = 310, R^2^ = 0.0002, *p* = 0.8006). (Aii) Histogram showing frequency distribution of 0 h dGAE and dGAE-488 species diameter. (Bi) Lengths of 72 h dGAE and dGAE-488 fibrils presented as a scatter column chart of all the values (N = 151 fibrils analysed for each condition). An unpaired t-test shows no significant difference in length of fibrils between 72 h dGAE (95.12 ± 4.28 μm) and 72 h dGAE-488 (84.96 ± 3.93 μm) (t = 1.748, df = 300, R^2^ = 0.01, *p* = 0.0815). (Bii) Histogram showing frequency distribution of 72 h dGAE and dGAE-488 fibril lengths. An unpaired t-test shows a significant difference between 0 h dGAE and 72 h dGAE (t = 16.17, df = 305, *p* < 0.0001) and between 0 h dGAE-488 and 72 h dGAE-488 (t = 15.22, df = 305, *p* <0.0001).

***Figure S2. Increasing concentrations of soluble forms of dGAE show no effect on cell death.*** Soluble (0 h) dGAE species (1-20 µM) were added to cells and left to incubate for 24 h. The percentage of buffer-treated cell death was quantified. Data shown are averages from six fields of view from 4-6 independent experiments ± SEM. A one-way ANOVA with Dunnett’s multiple comparison test showed no significant difference between buffer-treated cells (100 ± 3.02%) and cells treated with varying concentrations of dGAE (1 µM: 120.6 ± 7.86%, 2 µM: 122.3 ± 14.92%, 4 µM: 110.6 ± 10.71%, 6 µM: 130.1 ± 9.43%, 8 µM: 115.2 ± 11.08%, 10 µM: 114.9 ± 12.45%, 20 µM: 112.6 ± 16.33%).

***Figure S3. Comparable cell death effects between dGAE and dGAE-488.*** dGAE and dGAE-488 (100 µM) were agitated for 72 h. Soluble (0 h) or agitated (72 h) species (1 µM) was added to cells and left to incubate for 24 h. (A) Representative widefield images following exposure to buffer, dGAE or dGAE-488 with ReadyProbes^®^ reagent, showing total nuclei in blue and nuclei of dead cells in green (dGAE) or red (dGAE-488). Scale bar: 100 µm (B) The percentage of buffer-treated cell death was quantified for all conditions. Data shown are averages from six fields of view from 2 independent experiments ± SEM. A one-way ANOVA shows a significant difference between groups (F(4, 73) = 4.325, R^2^ = 0.1916, *p* = 0.0034). Dunnett’s multiple comparisons shows a significant difference between cells treated with buffer only (100 ± 5.77%) and cells treated with dGAE fibrils (72 h) (146.3 ± 6.63%) (*p* = 0.0117) or dGAE-488 fibrils (72 h 488) (143 ± 11.07%) (p = 0.0215) but not between buffer-treated cells and soluble dGAE treated cells (100 ± 7.88%) or soluble dGAE-488-treated cells (115.8 ± 15.18%).


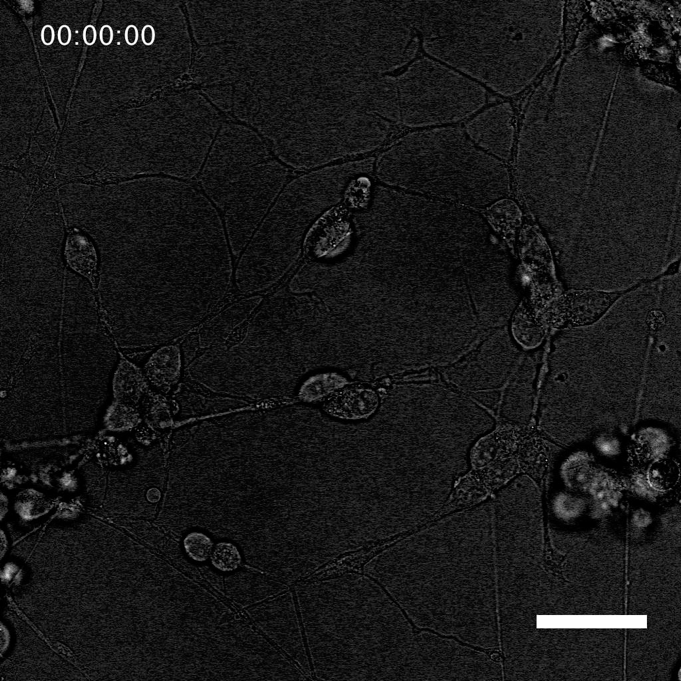


***Figure S4. Live-cell imaging movie following the addition of 1 µM soluble dGAE-488.*** Internalisation of soluble dGAE-488 (5 µM, unagitated) internalisation was monitored live by confocal microscopy from t = 0-14 h. Internalisation was observed from 2 h following the addition of dGAE-488. Scale bar: 50 µm.
